# Cytoskeleton-associated protein 5 and clathrin heavy chain binding regulates spindle assembly in mouse oocytes

**DOI:** 10.18632/oncotarget.15097

**Published:** 2017-02-04

**Authors:** Angeleem Lu, Cheng-Jie Zhou, Dong-Hui Wang, Zhe Han, Xiang-Wei Kong, Yu-Zhen Ma, Zhi-Zhong Yun, Cheng-Guang Liang

**Affiliations:** ^1^ The Key Laboratory of National Education Ministry for Mammalian Reproductive Biology and Biotechnology, The Research Center for Laboratory Animal Science, College of Life Science, Inner Mongolia University, Inner Mongolia, Peoples Republic of China; ^2^ Inner Mongolia Peoples Hospital, Hohhot, Inner Mongolia, Peoples Republic of China

**Keywords:** CKAP5, CLTC, oocyte, meiosis, spindle, Pathology Section

## Abstract

Mammalian oocyte meiotic maturation is the precondition of early embryo development. Lots of microtubules (MT)-associated proteins participate in oocyte maturation process. Cytoskeleton-associated protein 5 (CKAP5) is a member of the XMAP215 family that regulates microtubule dynamics during mitosis. However, its role in meiosis has not been fully studied. Here, we investigated the function of CKAP5 in mouse oocyte meiotic maturation and early embryo development. Western blot showed that CKAP5 expression increased from GVBD, maintaining at high level at metaphase, and decreased after late 1-cell stage. Confocal microscopy showed there is no specific accumulation of CKAP5 at interphase (GV, PN or 2-cell stage). However, once cells enter into meiotic or mitotic division, CKAP5 was localized at the whole spindle apparatus. Treatment of oocytes with the tubulin-disturbing reagents nocodazole (induces MTs depolymerization) or taxol (prevents MTs depolymerization) did not affect CKAP5 expression but led to a rearrangement of CKAP5. Further, knock-down of CKAP5 resulted in a failure of first polar body extrusion, serious defects in spindle assembly, and failure of chromosome alignment. Loss of CKAP5 also decreased early embryo development potential. Furthermore, co-immunoprecipitation showed that CKAP5 bound to clathrin heavy chain 1 (CLTC). Taken together, our results demonstrate that CKAP5 is important in oocyte maturation and early embryo development, and CKAP5 might work together with CLTC in mouse oocyte maturation.

## INTRODUCTION

Spindles are highly dynamic biomechanical structures assembled by microtubules (MTs) during mitosis and meiosis, and they play a crucial role in maintaining genome stability. In mammals, oocytes generate a haploid set of chromosomes by undergoing a single round of DNA replication, followed by two rounds of chromosome segregation. During this process, the meiotic spindle assembles around metaphase chromosomes and then moves peripherally to the cortex in an actin filament-dependent process [1]. With the completion of the chromosome segregation, spindle depolymerize into cytoplasm. Lots of molecules involve into the spindle assembly in oocyte meiosis. The microtubule-associated protein mini-spindles (Msps) is recruited to spindle poles by Kinesin-14 [2], where it prevents loss of bipolarity possibly by stabilization of MTs ends [3], then ZYG-9 is enriched at spindle poles and required for spindle assembly [4]. Proper structure and function of spindles ensure faithful chromosome segregation. Numerous MT-associated proteins (MAPs) participate in regulating spindle organization [5–7], including cytoskeleton-associated protein 5 (CKAP5, also named colonic and hepatic tumor over-expressed gene (ch-TOG) in humans). CKAP5 belongs to the *Xenopus* MAP 215 (XMAP215) family [8]. Members in this family include XMAP215 in *Xenopus* [9], DdCP224 in *Dictyostelium* [10], STU2 in budding yeast [11], Dis1 and its second homologue Alp14 in fission yeast [12, 13], ZYG-9 in *Caenorhabditis elegans* [14], Msps in *Drosophila* [15], ch-TOG in humans, and MOR1 in *Arabidopsis* [16, 17]. These proteins have highly conserved structures and are characterized by tumor overexpressed gene (TOG)-containing domains with HEAT motifs in the N-terminal [18, 19].

A previous study reported that XMAP215 acts as a processive polymerase at the MT plus-end to accelerate MT assembly [20]. In *Drosophila*, Msps localizes to the acentriolar pole and is required for maintaining meiotic spindle bipolarity [3]. STU2 plays a critical role in regulating kinetochore-derived MTs and promotes MT assembly by accelerating MT growth and preventing catastrophe [11, 21]. In general, XMAP215 family members participate in the regulation of MT dynamics during mitosis despite their different subcellular localizations.

Ch-TOG, which was discovered in several types of tumor tissues, is abundantly expressed during mitosis [22]. A recent report showed that ch-TOG is recruited by SLAIN1/2, two MT plus-end tracking proteins, thereby promoting MT growth. Disrupting the SLAIN-ch-TOG complex increases catastrophe frequency and inhibits axon elongation during neuronal development [23].

In human somatic cells, ch-TOG protein is strongly concentrated at spindle poles and is essential for spindle pole organization, centrosome integrity, and spindle bipolarity [24]. Depletion of ch-TOG reduces spindle MT turnover and reduces chromosome oscillations [25]. A complex of transforming acidic coiled coil 3 (TACC3)/ch-TOG/clathrin, the shortest class of inter-MT bridge of kinetochore-fibers (K-fibers), is required for stabilizing MTs in the mitotic spindle [26, 27]. Specific removal of TACC3/ch-TOG/clathrin results in a loss of a subpopulation of inter-MT bridges and reduces K-fiber tension [28].

The role of CKAP5 in mammalian meiotic progression has not been examined. In a previous study, we showed that clathrin heavy chain 1 (CLTC) plays an important role in spindle assembly and chromosome congression *via* an MT-related mechanism during mouse oocyte maturation [29]. In the current study, we investigated the expression and function of CKAP5 during oocyte meiotic maturation and early embryo development and explored the relationship between CKAP5 and CLTC in meiotic spindle assembly and organization.

## RESULTS

### Expression and subcellular localization of CKAP5 during mouse oocyte meiotic maturation and early embryo development

**Figure 1:**
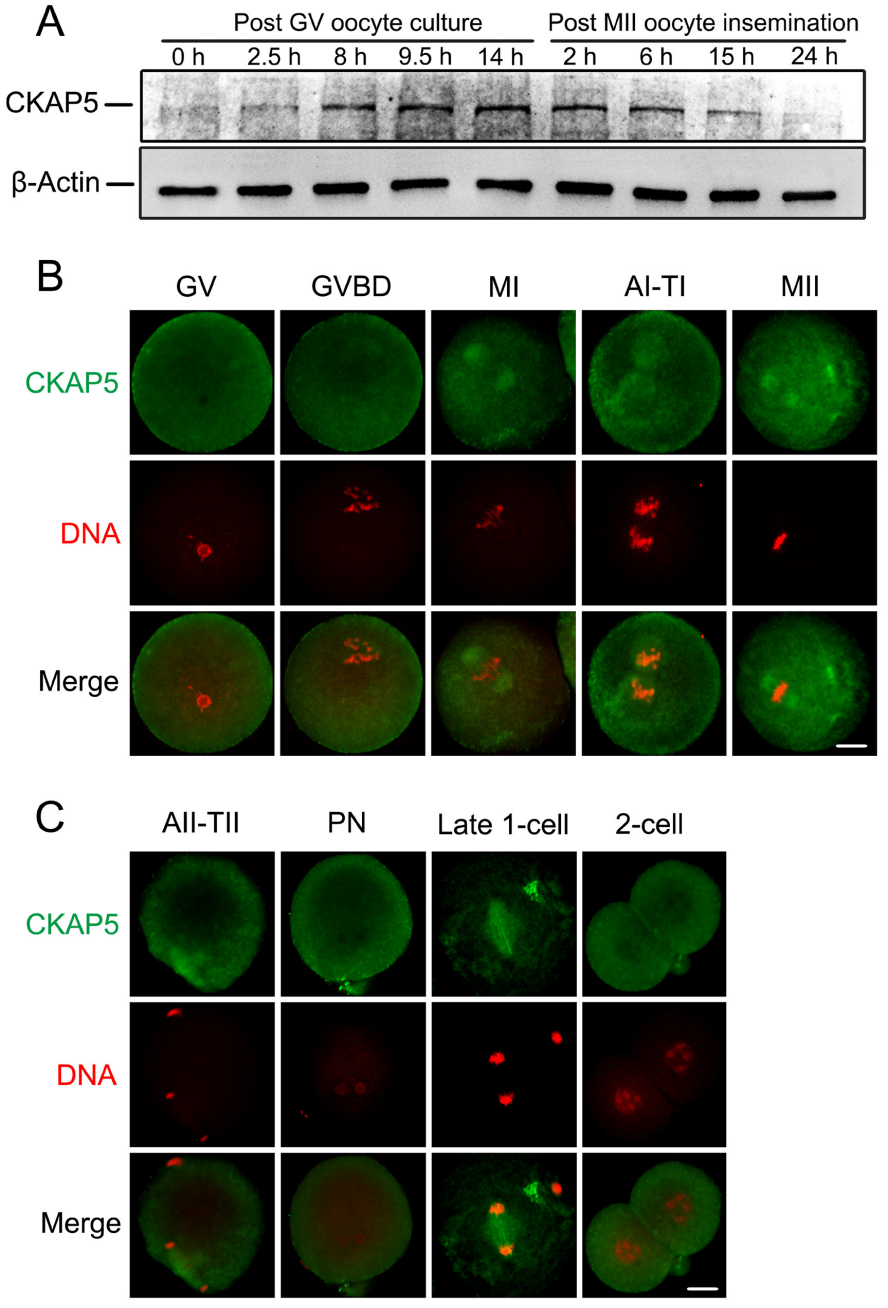
Expression and localization of CKAP5 during oocyte meiotic maturation and early embryonic development **A**. Western blot analysis of CKAP5 in oocyte and early embryonic developmental stages. A total of 200 mouse oocytes or embryos per sample were used. β-actin was used as a loading control. Experiments were performed in triplicate. The results of one representative experiment are shown. **B**. and **C**. Localization of CKAP5 (green) during each stage of oocyte and early embryo development as detected by immunofluorescence staining. DNA was stained with PI (red). Slides were examined using a confocal microscope. Scale bar, 20 μm.

We first examined the expression and subcellular localization of CKAP5 during mouse oocyte meiotic maturation and early embryo development. Western blot analysis detected CKAP5 at 0, 2.5, 8, 9.5 and 14 hours after GV culture, and 2, 6, 15, and 24 hours after MII oocyte insemination. (Figure 1A). CKAP5 expression was relatively low at the germinal vesicle (GV) stage, gradually increased and reached its highest level at metaphase II (MII), remained high through the pronuclear (PN) stage, and decreased during late 1-cell and 2-cell stages.

Immunofluorescence and confocal microscopy showed no specific accumulation of CKAP5 in GV oocytes (Figure 1B). After GV breakdown (GVBD), CKAP5 accumulated around the chromosomes in the spindle area. At MI and MII stages, CKAP5 concentrated around the aligned chromosomes, putatively the position of the metaphase spindle. As oocytes proceeded to AI-TI and AII-TII phases, CKAP5 organized into a bipolar structure when homologous chromosomes or sister chromatids were separated and migrated in opposite directions (Figure 1B and 1C). CKAP5 dispersed into the whole cytoplasm upon PN formation and re-accumulated between the separating chromosomes during the first mitosis of zygotes. CKAP5 distributed diffusely again in the interphase of 2-cell-stage embryos (Figure 1C). Briefly, the subcellular localization pattern of CKAP5 displayed cell cycle dependent manner in accordance with spindle dynamics.

### Localization but not expression of CKAP5 is disturbed by spindle-perturbing agents in metaphase oocytes

**Figure 2:**
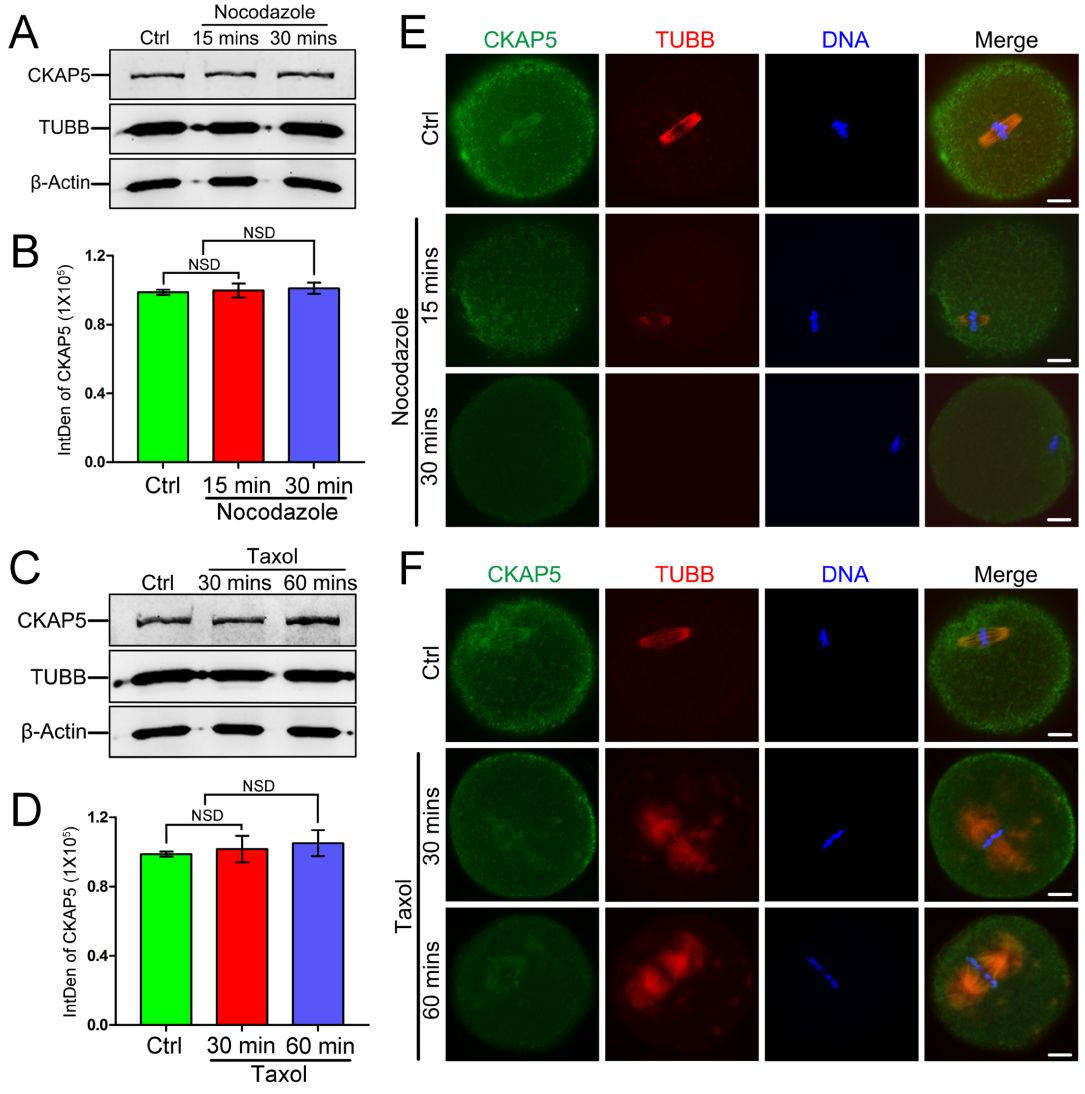
Expression and localization of CKAP5 in mouse oocytes treated with nocodazole or taxol Western blot and densitometry of CKAP5 from oocytes treated with nocodazole **A**., **B**. or taxol **C**., **D**. β-actin was used as a loading control. Experiments were performed in triplicate. The results of one representative experiment are shown. Confocal images of CKAP5 (green), TUBB (red), and DNA (blue) after treatment with nocodazole **E**. or taxol **F**. Scale bar, 10 μm.

To further investigate the relationship between CKAP5 and MTs, we treated MII oocytes with the spindle-perturbing agents nocodazole and taxol. Western blot analysis showed that nocodazole treatment for 15 or 30 minutes did not affect the amount of CKAP5 or tubulin beta class I (TUBB; Figure 2A and 2B). Similar results were obtained after taxol treatment for 30 or 60 minutes (Figure 2C and 2D). In oocytes without drug treatment, CKAP5 mainly co-localized with TUBB. However, nocodazole treatment resulted in diffuse CKAP5 expression and gradually depolymerized MTs (Figure 2E). By contrast, taxol caused excessive polymerization of MTs and formation of asters in the spindle architecture, accompanied by a similar distribution of CKAP5 (Figure 2F). These results showed CKAP5 is associated with MT dynamics during depolymerization and polymerization.

### Knock-down of CKAP5 at the GV stage causes spindle disassembly and failure of chromosome congression in oocyte maturation

**Figure 3:**
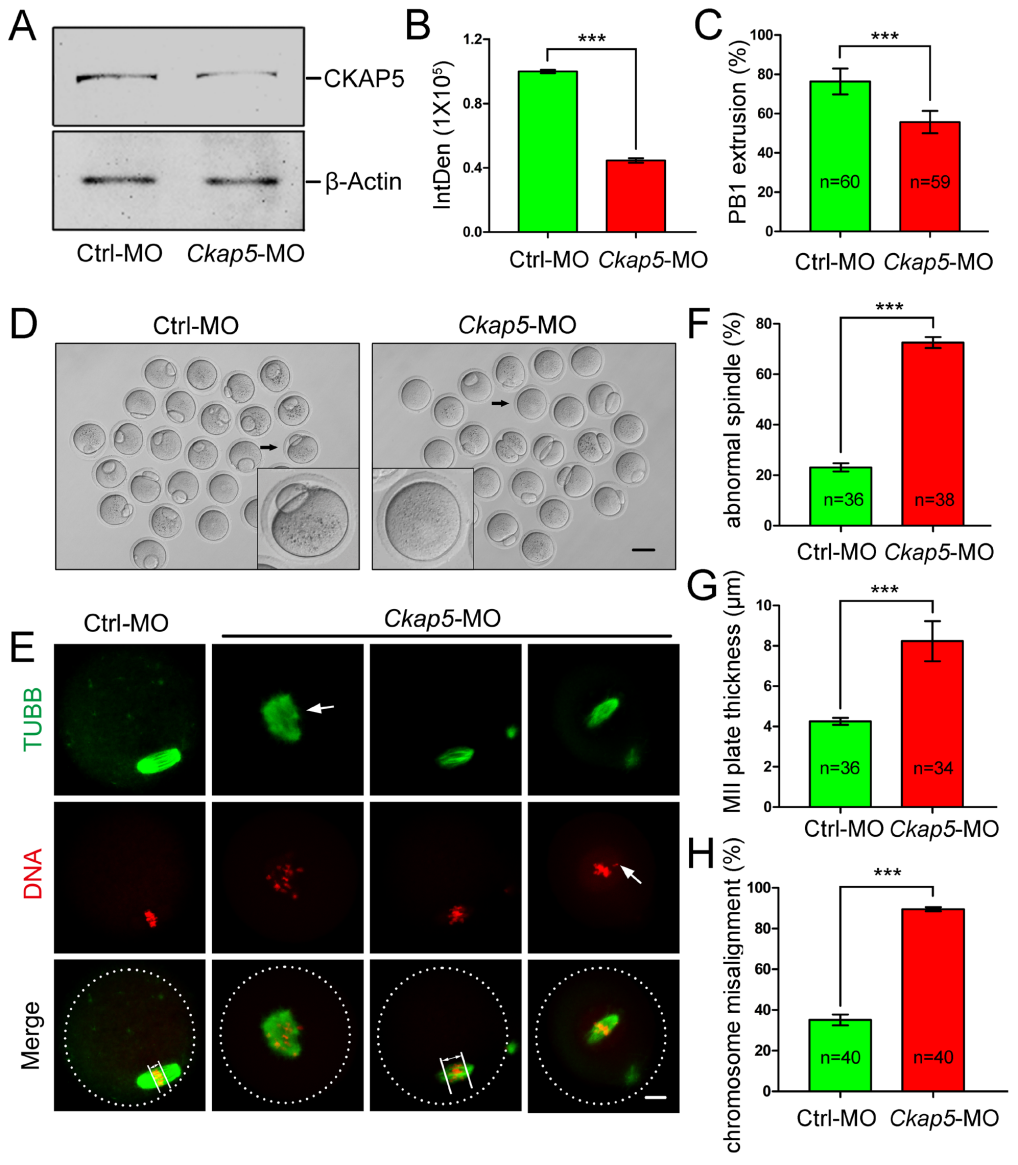
Knock-down of CKAP5 ***via*** MO injection causes failure of spindle assembly and chromosome congression. **A**. Western blot and **B**. densitometry of CKAP5 expression after control or *Ckap5* MO injection. β-actin was used as a loading control. The results of one representative experiment are shown. **C**. and **D**. CKAP5 knock-down decreased PB1 extrusion. The black arrows indicate a representative oocyte after control or *Ckap5* MO injection. Scale bar, 50 μm. **E**. Confocal images of MO-injected GV-stage oocytes 14 hours after release from milrinone. Control oocytes exhibited normal spindles and chromosome congression (left column), whereas oocytes injected with *Ckap5* MO displayed various abnormal spindle-chromosome complexes (right three columns). Arrows in the first and third columns indicate the width of the MII plate. The arrow in the second column indicates an abnormal spindle. The arrows in the fourth column indicate dispersed chromosomes. White frames indicate the edges of oocytes. Oocytes were immunostained for TUBB (green) and DNA (red). Scale bar, 10 μm. **F**. CKAP5 knock-down caused spindle abnormalities. **G**. CKAP5 knock-down increased the thickness of MII plates. **H**. CKAP5 knock-down caused chromosome misalignment. Data are presented as the mean ± SD of at least three independent experiments. ***P < 0.001, Student's *t*-tests.

We next microinjected *Ckap5* morpholino (MO) into GV oocytes to knock-down the expression of CKAP5. Western blot analysis (Figure 3A) and subsequent densitometry (Figure 3B) showed decreased CKAP5 expression in the *Ckap5* MO-injected group compared with the control group (*P* < 0.001), demonstrating the efficiency of CKAP5 knock-down *via* MO injection. We then tracked first polar body (PB1) extrusion 16 hours after release of milrinone. CKAP5 knockdown significantly reduced the percentage of oocytes that released their PB1 (*P* < 0.001; Figure 3C and 3D). Moreover, severe spindle assembly and chromosome congression defects were observed in *Ckap5* MO-injected oocytes (Figure 3E). These defects were classified into three types. The first type was a failure in the development of fusiform spindle poles (Figure 3E, second column). The second type was the formation of a thick MII plate (Figure 3E, third column), which has been reported as the test index of oocyte quality [30], appearing as a longer distance between the edges of the chromosomes perpendicular to the spindle axis. The third type was a failure of chromosome segregation (Figure 3E, fourth column) due to an inability of the spindle to direct chromosomes to separate properly. We quantified the percentage of abnormal spindles, MII plate thickness, and the percentage of misaligned chromosomes. All measures indicated that *Ckap5* MO-injected oocytes had abnormal spindle-chromosome complexes compared with control oocytes (Figure 3F, 3G, and 3H, *P* < 0.001).

### Knock-down of CKAP5 in the PN stage impairs early embryo development

**Figure 4:**
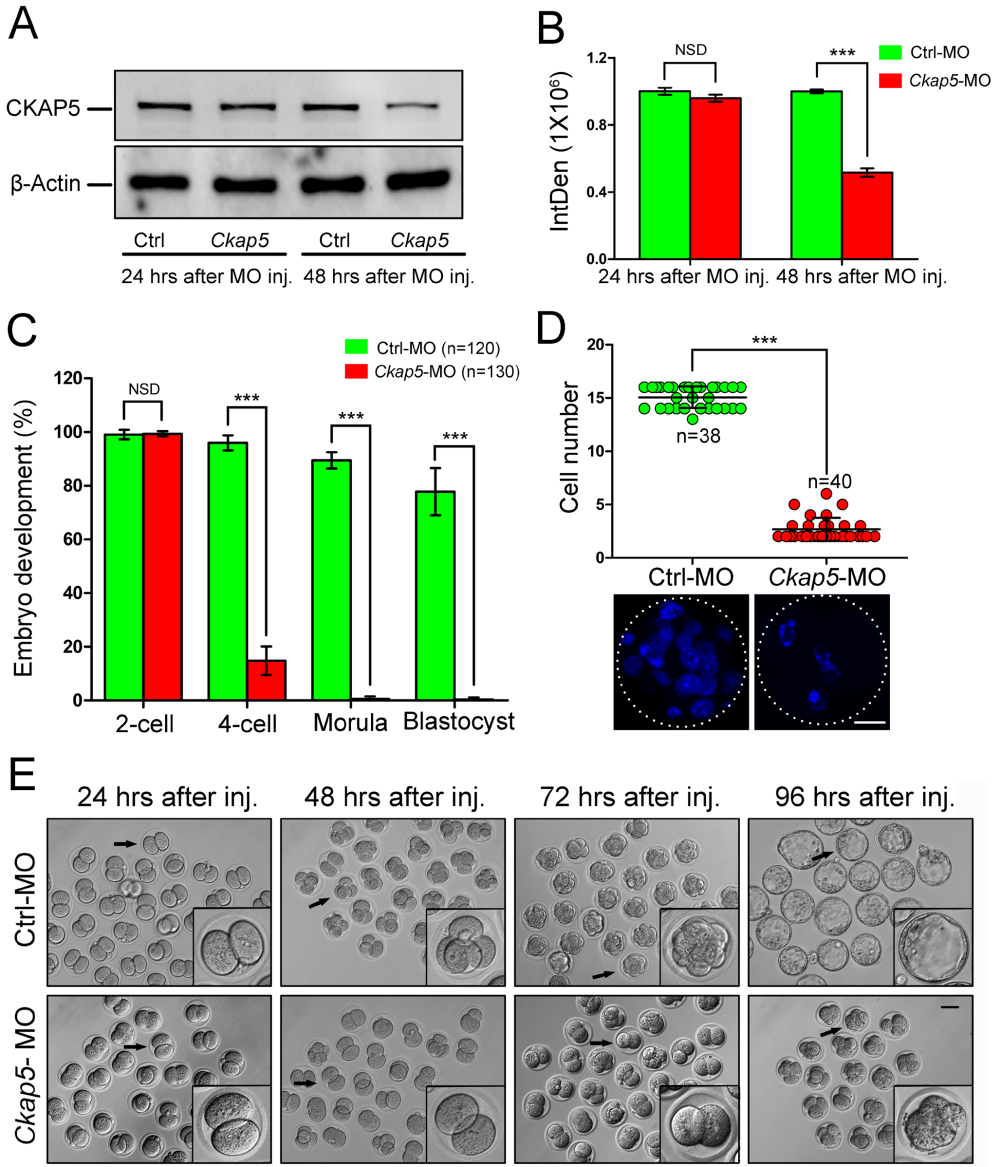
Knock-down of CKAP5 in the PN stage impairs embryonic development **A**. Western blot analysis and **B**. densitometry of CKAP5 expression after control or *Ckap5* MO injection into PN-stage embryos after 24 or 48 hours of culture. β-actin was used as a loading control. **C**. CKAP5 knock-down affected early embryo development. Oocytes at the PN stage were injected with control or *Ckap5* MO, and embryo developmental rate was measured. **D**. Cell number in embryos derived from control or *Ckap5* MO groups 72 hours after injection. Each dot represents one zygote injected with control (green) or CKAP5 (red) MO. White frames indicate the edges of embryos. Scale bar, 20 μm. **E**. Representative images of early embryos 24, 48, 72, and 96 hours after control or *Ckap5* MO injection. Representative embryos (black arrows) are shown enlarged. Scale bar, 50 μm. Data are presented as mean ± SD of at least three independent experiments. NSD, no significant difference. ***P < 0.001, Student's *t*-tests.

To further demonstrate whether knock-down of CKAP5 affects embryo development, oocytes were injected with *Ckap5* MO within 1 hour after PN formation. Embryos were collected 24 or 48 hours after microinjection. We found that CKAP5 expression was not reduced after 24 hours of culture in the *Ckap5* MO-injection group (*P* > 0.05). However, a significant decrease was detected after 48 hours of culture (*P* < 0.001; Figure 4A and 4B).

No significant difference in the percentage of 2-cell-stage embryos was observed between control and *Ckap5* MO-injected groups (*P* > 0.05; Figure 4C). However, CKAP5 knockdown significantly reduced the percentage of 4-cell-stage embryos (*P* < 0.001; Figure 4C). Moreover, cell numbers in the *Ckap5* MO-injected group were reduced after 72 hours of culture (*P* < 0.001; Figure 4D). Population-based images of blastocysts illustrate the smaller numbers of *Ckap5* MO-injected embryos progressing through development, with many abnormal embryos exhibiting fragmentation and 2-cell-stage arrest (Figure 4E). Collectively, these results indicate that knock-down of CKAP5 in early embryos resulted in abnormalities and impairment of embryo development.

### CKAP5 and CLTC binding regulates spindle formation during oocyte meiosis

**Figure 5:**
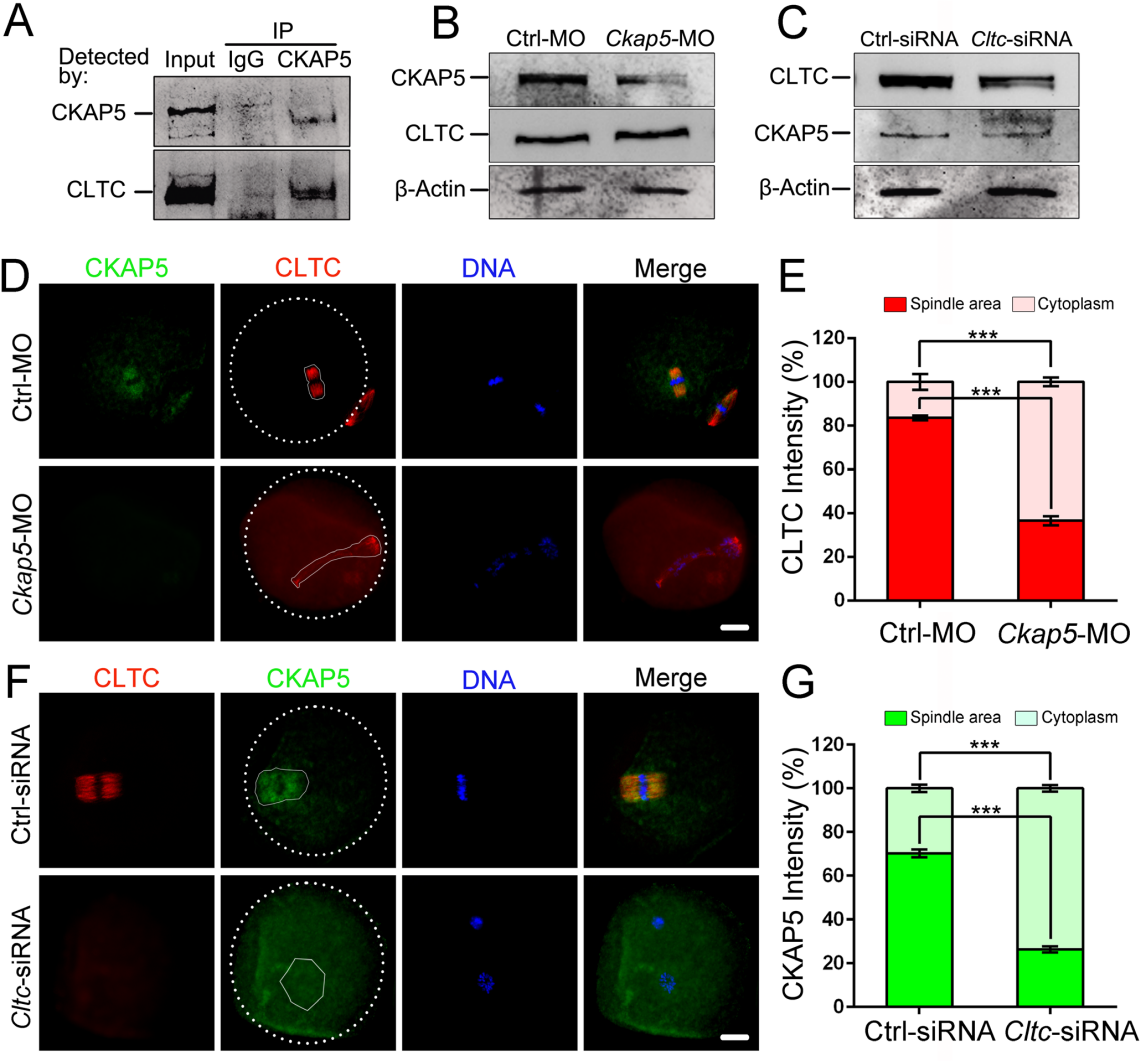
CKAP5 binds to CLTC during meiotic maturation **A**. CKAP5 binds to CLTC in oocytes. Co-immunoprecipitation was performed to assess the interaction between CKAP5 and CLTC. The blots of eluates were probed with anti-CKAP5 and anti-CLTC antibodies. **B**. Western blot analysis showing levels of CKAP5 and CLTC proteins from meiotic extracts of oocytes after CKAP5 knock-down. β-actin was used as a loading control. **C**. Western blots showing the levels of CLTC and CKAP5 proteins from meiotic extracts of oocytes after CLTC knock-down. β-actin was used as a loading control. **D**. CKAP5 knock-down affects CLTC distribution in MII-stage oocytes. Oocytes were immunostained for CKAP5 (green), CLTC (red) and DNA (blue). White full lines indicate the edge of spindle area. White dashed lines indicate the edge of oocyte. Scale bar, 20 μm. **E**. Intensity of CLTC in the spindle and cytoplasm area after *Ckap5* MO injection in MII-stage oocytes. **F**. CLTC knock-down affects CKAP5 distribution in MII-stage oocytes. Oocytes were immunostained for CLTC (red), CKAP5 (green) and DNA (blue). White full lines indicate the edge of spindle area. White dashed lines indicate the edge of oocyte. Scale bar, 20 μm. **G**. Intensity of CKAP5 in the spindle and cytoplasm area after *Cltc* siRNA injection in MII-stage oocytes. Data are presented as mean ± SD of at least three independent experiments. ***P < 0.001, Student's *t*-tests.

Our previous study showed that CLTC plays an important role in spindle assembly and chromosome congression [29]. The dynamic subcellular localization of CLTC is similar to that of CKAP5, suggesting that CKAP5 may interact with CLTC in mammalian meiotic oocytes. We performed co-immunoprecipitation assay followed by western blot analysis to detect a protein-protein interaction between CKAP5 and CLTC. We found that CLTC was pulled down by CKAP5, indicating their direct interaction in mouse oocytes (Figure 5A).

To further elucidate the interdependence between CKAP5 and CLTC in spindle organization, we first detected CLTC expression in CKAP5-depleted oocytes. Knock-down of CKAP5 by MO had no influence on the level of CLTC expression (Figure 5B). Likewise, knock-down of CLTC did not affect the level of CKAP5 expression (Figure 5C).

Next, we assessed the distribution of CLTC in CKAP5-dysfunctional oocytes. The amount of red fluorescence was measured by detecting the intensity in whole area of spindle or cytoplasm. In normal oocytes, CLTC or CKAP5 accumulated around the chromosome in a relatively concentrated spindle area (Figure 5D, first line). However, the localization of CLTC was disturbed in CKAP5 dysfunctional oocyte (Figure 5D, second line). The result of intensity detection showed the amount of CLTC was less in the spindle area than in the cytoplasm (*P* < 0.001; Figure 5E), which indicates that CLTC diffused from the spindle area to the peripheral cytoplasm upon CKAP5 knock-down. Likewise, dysfunction of CLTC disturbed the localization of CKAP5 (Figure 5F), indicating that CKAP5 diffused from the spindle area to the peripheral cytoplasm upon CLTC knock-down. (*P* < 0.001; Figure 5G).

## DISCUSSION

CKAP5 is an MAP that plays key roles in eukaryote spindle assembly, coordinating the activation of different mechanisms by directly coupling MT dynamics to spatial and temporal cues [31]. However, the molecular details of CKAP5 function in mammalian meiotic maturation remain poorly understood. In this study, we profiled CKAP5 expression and found that knock-down of CKAP5 disrupted oocyte meiotic spindle organization and chromosome alignment and impaired the embryo developmental potential of mouse oocytes. We further demonstrated a relationship between CKAP5 and another component of inter-MT bridges, CLTC, and their role in regulating spindle assembly and chromosome congression.

Our observation of CKAP5 localization patterns in oocytes differs from reports that other family members, such as Msps in *Drosophila* and ch-TOG in HeLa cells, are mainly localized to the centrosome and contribute to spindle pole integrity and organization in mitotic cells [32, 33]. In *Xenopus* oocyte, XMAP215 localized to the spindle during meiosis metaphase [9]. In our study, we observed that CKAP5 localized to the spindle in mouse oocyte meiosis, which is similar to *Xenopus* oocyte. This may be explained by the difference of cell types and species. However, in mouse oocytes, whether CKAP5 participates in spindle pole organization still needs further investigation.

A relationship between CKAP5 and MTs was further confirmed by experiments employing nocodazole and taxol treatment. In MII-stage oocytes, nocodazole treatment led to the disappearance of CKAP5 from the spindle and spindle collapse. Similar results were found in HeLa cells, demonstrating that ch-TOG binding with centrosome can regulate centrosome integrity and spindle pole organization [24]. As vertebrate oocytes contain acentriolar microtubule organizing centers (MTOCs) that may substitute centrosomes during acentrosomal spindle assembly [34], we propose that CKAP5 participates in MT organization. Thus, disruption of MT organization leads to disordered CKAP5 localization. Indeed, we found that CKAP5 was localized to cytoplasmic asters (which are characteristic for mouse oocytes but not for other mammalian oocytes) after taxol treatment. This is consistent with previous research showing that ch-TOG is required for mammalian MT aster assembly in a taxol-treated cell-free system [35].

We found that CKAP5-depleted oocytes exhibited severe defects in meiotic spindle organization and chromosome alignment. Previous studies demonstrated that CKAP5 regulates MT assembly and that CKAP5 knock-down results in a loss of spindle pole focus and reduces spindle MT density with slower MT turnover [24, 25, 36]. Similarly, meiotic spindles in *Xenopus* egg extracts become shorter upon disruption of XMAP215 [37], as XMAP215 is a processive polymerase at the MT plus-end that accelerates MT growth [20]. XMAP215 depletion leads to shorter spindles or defects in spindle morphology in *Schizosaccharomyces pombe, Caenorhabditis elegans, Xenopus* egg extracts*, Drosophila melanogaster* and HeLa cells [38]. But in our study, we did not observe the shorter spindles, probably due to different species and cell types. Furthermore, the major functions of TOGp (the human homolog of XMAP215/Dis1) during mitosis are to focus MT minus ends at spindle poles, maintain centrosome integrity, and contribute to spindle bipolarity [24]. The misalignment of chromosomes reflects a missing connection between MTs and kinetochores at the metaphase plate. Previous reports showed that CKAP5 regulates tension between sister kinetochores to control chromosome mobility but is not required for the formation of connections between MTs and kinetochores [25, 36]. In CKAP5-depleted somatic cells, kinetochore oscillations were severely depressed, and kinetochores moved very short distances and were unable to sustain mobility in either direction [25]. Therefore, we speculate that CKAP5 is involved in chromosome segregation during meiotic maturation.

We also found that CKAP5 was essential for mouse early embryo development. Embryos with CKAP5 knock-down could not progress past the 2-cell stage to achieve higher levels of development, even if embryos were cultured for a longer time. This indicates that CKAP5 knock-down severely affects embryo development and may prevent developmental progression. As the mechanism of cell division at the early embryo stage is similar to that for somatic mitotic division, we speculate that the role of CKAP5 in early embryo spindle assembly relies on a relationship with MTs. A recent study reveals that a structurally diverse yet positionally conserved TOG array in different family members drives MT polymerization and that the TOG domain of human ch-TOG engages with α-tubulin [19]. A series of HEAT motifs in TOG domains form a 60-Å oblong structure and this highly conserved intra-HEAT loops on one face is confirmed to be the tubulin-interaction surface [18, 39]. And mutations of the conserved residues in these loops abolish binding to the tubulin dimer [40]. The XMAP215 and another MAPs, CLASP families bind to soluble tubulin dimers to regulate MT dynamics using TOG domains [40–42]. The common signature of all members of this family is an N-terminal repeating structure consisting of TOG domains with HEAT motifs [19, 32]. Stretches of positively charged residues from the C-terminal to the TOG domain mediate direct MT lattice binding activity [20]. Thus, CKAP5-depleted embryos cannot develop further without normal MT organization.

Our previous study showed that CLTC regulates spindle assembly and chromosome congression during mouse oocyte meiotic maturation. The phenotypes of defects in spindle assembly and chromosome alignment after knock-down of CLTC are similar to those of CKAP5-depleted oocytes [29]. CKAP5 and clathrin form a TACC3/ch-TOG/clathrin complex that contributes to the stabilization of K-fibers in mitotic cells [26, 43, 44]. We hypothesize that CKAP5 also regulates spindle assembly and chromosome congression in oocytes by forming TACC3/ch-TOG/clathrin complexes.

TACC and clathrin interact directly when TACC is phosphorylated by aurora A kinase [44, 45]. A further study showed that the N-terminal domain of clathrin coordinates with TACC3 to load the complex onto MTs [46]. Consistent with findings in mitotic cells [44], we found that CLTC co-immunoprecipitated with CKAP5 in MII-stage oocytes. Thus, CKAP5 and CLTC interacted and formed a complex in mouse oocytes. Moreover, we found that the amounts of CKAP5 or CLTC protein were not affected by knock-down of each other. However, the binding of CKAP5 to spindle MTs was reduced after CLTC knock-down, resulting in an increase of CKAP5 in the cytoplasm. Similarly, CKAP5 knock-down caused abnormal CLTC distribution. These results are consistent with findings that recruitment of CLTC to the spindle is reduced in CKAP5-depleted somatic cells [26]. Thus, the mechanism by which CKAP5 and CLTC are recruited to MTs in meiotic oocytes needs further investigation.

In conclusion, our results suggest that CKAP5 plays a pivotal role in regulating spindle assembly and chromosome congression during mouse oocyte meiotic maturation, and its function may depend on binding with CLTC.

## MATERIALS AND METHODS

### Ethics statement

All studies adhered to procedures consistent with the National Research Council Guide for the Care and Use of Laboratory Animals and were approved by the Institutional Animal Care and Use Committee of Inner Mongolia University.

### Oocyte collection

Adult female (B6D2) F1 mice (4-8 weeks of age) were used for oocyte collection. GV-stage oocytes were collected 48 hours after injection of 5 IU pregnant mare serum gonadotropin (PMSG; SanSheng, Ningbo, China) by puncturing the follicles of ovaries. GV-, GVBD-, MI-, AI-TI-, and MII-stage oocytes were collected after culturing GV-stage oocytes for 0, 2.5, 8, 9.5, and 14 hours, respectively. AII-TII-, PN-, late 1-cell-, and early 2-cell-stage embryos were obtained after insemination for 2, 6, 15, and 24 hours, respectively. For *in vivo* MII-stage oocyte collection, mice were superovulated by injection of 5 IU PMSG followed by injection of 5 IU human chorionic gonadotropin (SanSheng) 48 hours later. Cumulus cells were dispersed by 0.3 mg/mL hyaluronidase in HEPES-M2 medium.

### *In vitro* fertilization and embryo culture

Adult male (B6D2) F1 mice (12-14 weeks of age) were used for sperm collection. The sperm suspension was capacitated for 2 hours in T6 medium supplemented with 10 mg/ml bovine serum albumin (BSA). MII-stage oocytes were incubated with spermatozoa for 6 hours in 200 μL T6 medium supplemented with 20 mg/mL BSA. Fertilized oocytes were cultured in CZB medium and transferred to CZB medium supplemented with 5.5 mmol/L glucose upon reaching the 4-cell stage. Oocytes and early embryos were cultured in a humidified atmosphere of 5% CO_2_ at 37°C. All chemicals and media were purchased from Sigma Aldrich (St. Louis, MO, USA) unless otherwise stated.

### Western blot analysis

Oocytes or embryos samples were lysed in Laemmli Sample Buffer (Bio-Rad Hercules, CA, USA). All other western blot procedures were conducted as previously described [47]. We used rabbit anti-CKAP5 (1:500, Pierce, Rockford, IL, USA), mouse anti-GAPDH (1:500, Proteintech, Chicago, IL, USA), mouse anti-β-Actin (1:200, Santa Cruz, Dallas, CA, USA), rabbit anti-CLTC (1:10,000, Abcam, Cambridge, MA, USA), and rabbit anti-β tubulin (1:500, Abcam) as primary antibodies. Perox-AffiniPure goat anti-Mouse IgG (H+L) (Jackson ImmunoResearch Laboratories, West Grove, PA, USA), Perox-AffiniPure donkey anti-Rabbit IgG (H+L) (Jackson ImmunoResearch Laboratories) were used for secondary antibodies. Bands on membranes were detected using an Enhanced Chemiluminescence Detection Kit (Pierce), and images were captured by Tanon-5200.

### Immunofluorescence

Oocytes were fixed in PBS-buffered 4% (v/v) paraformaldehyde (Electron Microscopy Sciences, Hatfield, PA, USA) for 50 minutes at room temperature followed by permeabilization with 0.5% (v/v) Triton X-100 for 2 hours. Oocytes were transferred to blocking buffer (PBS supplemented with 1% (w/v) BSA, 0.1% (v/v) Tween-20, and 0.01% (v/v) Triton X-100) for 1 hour and incubated with primary antibodies including rabbit anti-CKAP5 (1:100), mouse anti-β tubulin (1:1,000, Abcam), and rabbit anti-CLTC (1:1,000) overnight at 4°C. DyLightTM 488-conjugated AffiniPure Goat Anti-Mouse lgG + lgM (H+L), DyLightTM 549-conjugated AffiniPure Donkey Anti-Mouse lgG (H+L), Fluorescein (FITC)-conjugated AffiniPure Donkey Anti-Rabbit lgG (H+L) and DyLightTM 549-conjugated AffiniPure Donkey Anti-Rabbit lgG (H+L) were used as secondary antibodies (Jackson ImmunoResearch Laboratories). Oocytes were labeled with secondary antibodies for 1 hour. DNA was stained with 5 mg/ml propidium iodide (PI) or 4’,6-diamidino-2-phenylindole (DAPI, Roche, Mannheim, Germany).

### Drug treatment

For nocodazole treatment, nocodazole was prepared in dimethyl sulfoxide (DMSO) stock solution at 10 mg/mL and diluted in CZB medium to a final concentration of 10 μg/mL. Oocytes at MII stage were treated with nocodazole for 15 or 30 minutes and then fixed for immunostaining or lysed for western blotting. For taxol treatment, 5 mM taxol stock solution was prepared in DMSO and diluted in CZB medium to a final concentration of 10 μM. Oocytes at MII stage were treated with taxol for 30 or 60 minutes for immunostaining or western blotting. Control oocytes were treated in CZB supplemented with same concentration of DMSO.

### Microinjection of CKAP5 MO and Cltc siRNA

To assess CKAP5 function in mouse oocyte meiosis, CKAP5-specific MO (Gene Tool, Philomath, OR, USA) was used to knock down CKAP5 translation. Its nucleotide sequence was designed as 5’-CACTCACTGTCATCTCCCATTGTGC-3’. CKAP5 MO was diluted with RNase-free water to a final concentration of 1 mM. GV-stage oocytes were microinjected with 10 pL solution and incubated in CZB containing 2.5 μg/mL milrinone for 20 hours. Oocytes were washed thoroughly and transferred to fresh medium without milrinone for meiosis resumption. After an additional 14 hours in culture, MII stage oocytes were collected for analysis. The same amount of standard control MO 5’-CCTCTTACCTCAGTTACAATTTATA-3’ was injected into control oocytes under the same conditions. In CLTC knock-down experiments, Stealth RNAi ™ *Cltc* siRNA (Invitrogen, Shanghai, China) was diluted to a final concentration of 2 μM. Its nucleotide sequence was 5’-UAGAGGUGAAUAUAAUACUUGG-3’. Subsequent procedures were performed as described above.

### Measurements of MII plate thickness

MII plate thickness was measured by drawing two lines at the edges of PI staining and perpendicular to the spindle axis, defined by beta-tubulin staining. The distance between these two lines was used as the MII plate thickness. Each experiment was repeated at least three times.

### Immunoprecipitation

Approximately 1,000 oocytes at MII stage were collected and subject to co-immunoprecipitation using the Pierce™ Direct Magnetic IP/Co-IP Kit (Thermo Scientific, Rockford, IL, USA) following the manufacturer's instructions. Briefly, samples were added to immunoprecipitation lysis/wash buffer, and lysate was incubated on ice with periodic mixing. Pretreated rabbit anti-CKAP5 was coupled to NHS-activated magnetic beads on a rotating platform for 1 hour at room temperature. The antibody-coupled beads were collected on a magnetic stand and incubated with lysate solution overnight at 4°C. After removing unbound sample, beads were magnetically separated from target antigen through incubation in elution buffer for 5 minutes. Supernatant containing co-immunoprecipitation complex was processed for western blot analysis. As a negative control, antibody was replaced with non-related rabbit IgG (Equitech-Bio, Kerrville, Texas, USA).

### Statistical analysis

All experiments were performed at least three times. Data were analyzed by one-way ANOVA using SPSS 16.0 statistical software (International Business Machines Corporation, Armonk, New York, USA). Results are expressed as mean ± standard deviation (SD). In the figures, *P* < 0.05, *P* < 0.01, *P* < 0.001, and *P* > 0.05 are indicated by one asterisk (*), two asterisks (**), three asterisks (***), or no statistically significant difference (NSD), respectively.

## Abbreviations

GV, germinal vesicle; GVBD, GV breakdown; MII, metaphase II; CKAP5, cytoskeleton-associated protein 5; MO, morpholino; MT, microtubule; MAP, MT-associated protein; ch-TOG, colonic and hepatic tumor over-expressed gene; K-fiber, kinetochore-fiber; CLTC, clathrin heavy chain 1; PN, pronuclear; PB1, first polar body; PMSG, pregnant mare serum gonadotropin; BSA, bovine serum albumin; DMSO, dimethyl sulfoxide; XMAP215, *Xenopus* MAP 215; TACC3, transforming acidic coiled coil 3, SD, standard deviation.
